# Address1Read before the Virginia State Veterinary Medical Association, June 18, 1895.

**Published:** 1895-09

**Authors:** W. H. Harbaugh

**Affiliations:** President Virginia State V. M. A.; Richmond, Va.


### PRESIDENT'S ADDRESS.[1]

BY DR. W. H. HARBAUGH,
RICHMOND, VA.

In Virginia the veterinary profession is but in its infancy; the formation of this Association, we may say, marks the date of our profession in this State assuming anything like an independent or a distinct character, and, thanks to the members of our Association, the profession is now a healthy and rapidly developing infant. Ten years ago when I commenced to practise in Richmond there was no other graduate in that city; there were but two other graduates in the State. The first three veterinarians coming to Virginia remained but six months to two years. The fourth located at Norfolk, where he still practises. The fifth remained six months; others followed to remain a variable length of time.

[1] Read before the Virginia State Veterinary Medical Association, June 18, 1895.

In 1891 Dr. Niles came to Blacksburg, and soon after his arrival he wrote me in regard to forming a State Association, but after counting heads we could not discover a sufficient number of graduates in the entire State to officer a society properly, and there the matter rested, so far as we were concerned, for several years.

About the middle of January, 1894, a bill was introduced in the Legislature which had for its object the regulation of the practice of veterinary medicine and surgery. Parts of it I considered very objectionable, and I felt sure that it did not represent the desires of the majority of the graduates. I appeared before the legislative committee that had the bill to consider, and so vigorously opposed it that my objections were sustained by the committee and the bill was never reported. The chairman of the committee suggested that the veterinarians should agree upon a bill, and he would set a date satisfactory to all to have it considered. I immediately wrote all graduates I was aware of in the State; those I heard from reported others, and I was much surprised to learn that within a year the number had more than doubled. Dr. Niles again suggested the formation of an association, and urged that such a step was necessary either to secure the desired legislation or to block objectionable measures. Drs. Faville and Gilchrist proposed a conference at Richmond on the subject of legislation and notified me of the day they proposed to be there. I immediately telegraphed Dr. Niles, who responded in person.

On February 6, 1894, we agreed upon the minor details of a bill which the great majority of the graduates in the State had practically indorsed by letters. This bill was the one which simply asked for a legal definition and protection of our title.

After the legislative questions were settled, Dr. Faville proposed the organization of the Association, which was agreed to, and six of us signed the agreement. A temporary chairman and secretary were elected. A committee was appointed to formulate a charter and have the Association incorporated by act of Legislature. The chairman was authorized to call a meeting at such time as in his discretion was thought best after all preliminaries had been arranged.

The Committee on Charter concluded to adopt so much of the charter of the Medical Society of Virginia as was pertinent. The modified charter was presented to the Legislature and made a law, and this act incorporating our Association was the first law ever passed in this State which recognized the veterinary profession. When the bill to incorporate was presented we had eight members, and when the permanent organization took place we had twelve members.

On March 22, 1894, the first regular meeting was held at Richmond. It was a busy day for those who attended the meeting, and a red-letter day for the profession in this State. The whole time was occupied with regular business. No essays were read and no cases reported.

The bill we presented to protect and define our titles never became a law. Our next meeting was held at Norfolk, August 15, 1894. It was a successsful meeting in every respect, and the bountiful hospitality of the Norfolk members will ever be pleasantly remembered by all who were in attendance.

Dr. Faville read a paper on "Malaide du Coit," and demonstrated his description with pathological specimens taken from affected animals. This subject was a rare treat to veterinarians, and I think it scarcely possible for any one present at that meeting to fail to recognize the disease if met with in practice. Practical papers and demonstrations of this kind will do more to create interest and assure a full attendance than any other attractions we can offer. In the appointment of essayists for future meetings I hope this suggestion will be followed: At least one essayist should be selected who will read a paper on a surgical subject and demonstrate practically his method of operating. Other members may also be induced to operate, provided subjects are secured for them. This feature, if introduced, will have considerable bearing in the selection of places for holding meetings.

Dr. Niles read an exhaustive paper on "Tuberculosis," which showed much labor in its preparation, and was remarkable for its practical work. The paper was published and indorsed by medical and agricultural journals, and extracts were re-published by the daily press. It did much toward bringing the subject before the public in a manner creditable to both Dr. Niles and the profession. At this meeting one member was dropped and two new members were elected.

The next meeting was held at Charlottesville, January 3, 1895, and proved to be our most successful meeting in more respects than one. Papers on different subjects were read and discussed and interesting cases were reported. The Charlottesville members provided a regular Christmas-time entertainment for the visitors, and the whole time of our sojourn was profitably and enjoyably occupied. Six new members were elected at the Charlottesville meeting.

We are now at Blacksburg for the purpose of holding our first annual meeting, and the history of it must be made before it can be written. I can only say that I hope this meeting, as well as all future meetings, will be attended with the same good share of success as fell to our lot during the past year.

The affairs of the Association are in good condition. We have become a fixture, and it should be the aim of every member so to conduct his professional work that in time the public will recognize the fact that only legitimate veterinarians can be members.

So far as I know there are not more than five eligible men in the State who are not members or applicants for membership. This emphasizes the fact that we are not only the representative organization of our profession, but that we almost wholly constitute the profession in Virginia.

The more competent men who locate in the State, the sooner will the profession arrive at its proper standing beside other learned professions. Do not be afraid that new-comers will deprive us of our living, they only help to educate people up to the standard of employing legitimate men; they certainly help to create a demand. It is not many years since I was the only graduate in Richmond, now there are five, and I can honestly assure you that I now do more than five times as much business as I did when I had the field to myself, and I presume the other practitioners there are as well satisfied as I am.

There are some good places in the State without graduates, and I feel certain that proper men could build up good practices in them. But new-comers should be informed that it requires patience and perseverance to build up a paying and permanent practice, and that in a new place where people have been in the habit of employing different neighbors when their animals were ill they do not in all cases immediately desert those neighbors upon the arrival of a new man, even if he is a graduate in veterinary medicine. It takes time to prove to the people that the diploma carries with it more knowledge than is acquired by years of dosing animals.

As the profession grows in importance, young men of our State are looking toward it with a view of entering its ranks, and our advice is often sought in this regard. Our advice in such cases should be guarded; we should consider well the fitness of the individual who applies for advice. We are also asked for an opinion as to the best college, and here, again, our opinion should be guarded, and also qualified. There are colleges and colleges, but to pick out the best for all applicants is a difficult task. But some applicants do not want the best, they want to know the one easiest to get through, and I am sorry to say that we all must admit that there is at least a sufficient number of this kind to give the applicant an opportunity to select.

For some time past there has been much agitation in respect to the relative merits of the two-terms and three-terms veterinary schools. There can be no doubt that a properly equipped three-terms school can impart more knowledge and impart it better than a two-terms school similarly equipped, but we must not overlook the fact that some of the best veterinarians of our time were graduated from two-terms schools; however, their great advance over their contemporaries is to be credited in a large extent to their individual efforts rather than to their alma mater. We are all acquainted with men who might attend colleges five, ten, or even twenty years and still be unfit to practise; while we know others who were really competent men at the end of two years. This refers mainly to theoretical knowledge; but how about practical knowledge? In my opinion the question does not hinge so much on the number of years a man attends college as it does on the intelligence and the adaptability of the individual to acquire and utilize the special instruction imparted to him. The comprehension of men differs vastly. Some men can acquire more knowledge of a special character in a year than others can in a lifetime, and it would serve no good purpose to compel the quick, intelligent student to remain in college till the slow, dull student was sufficiently educated. At best, the course at colleges can only be considered necessarily preparatory; the real test is when one is brought face to face with the stern realities of practice, and many bright students make absolute failures as practitioners.

It is a notorious fact that the amount of practical instruction imparted at colleges is shamefully inadequate. Students are graduated who have never witnessed even one of the minor operations, and who have not had the benefit of any clinical demonstrations whatever.

The average professor enters the lecture hall, talks for an hour or less on a subject, quotes the conflicting opinions of different authors, and then departs, leaving the mind of the student in such a confused state that he is compelled to resort to his text-book to ascertain what the professor was talking about. Quite a number of these professors are totally unfit to teach on account of either not having a good understanding of the subject or from a lack of a certain tact which is necessary to impart knowledge to others, and some are medical doctors who have no practical knowledge of the animals we treat, but still they mount a platform and pretend to teach what they do not know themselves. The result of such methods of instruction cannot fail to discourage many students who, under more favorable methods, would become competent men.

Much has been said about insufficient examinations to ascertain the fitness of graduates, but if the examinations of students should be rigid, which we all must admit, how much more is it necessary that institutions should require a still more rigid examination before a man is given a professorship?

It is often urged that the advance in veterinary science has made three terms necessary, but while the advance in all scientific branches, which, as a whole, constitute veterinary science, has been great, it must be remarked that the facilities for acquiring and imparting knowledge have advanced in equal, if not greater, proportions, so that one can now gain much more exact knowledge in a given time than he formerly could. Many important questions that in former times were in doubt and darkness have been cleared up and are now thoroughly understood.

Simply because a school advertises three terms is not in itself sufficient to warrant us in indorsing it; we should know that the teachers are competent men, not failures in practice, as we know many of them to be; we should also know that the schools we indorse honestly devote a great part of each session to practical instruction. A man who professes to be a teacher should be competent to demonstrate practically to his class what he teaches.

One can scarcely peruse a college catalogue that does not contain a laudatory notice of the facilities for imparting practical knowledge, but consult the students from the various institutions and you will ascertain that but few of the schools live up to their advertisements.

All associations have, I believe, a code of ethics to govern the professional conduct of the members, and it strikes me as very inconsistent that we require our members not to do things we silently overlook in the advertising and other business methods of some colleges. We would condemn a practitioner who would solicit work from the client of a fellow-practitioner, and rightly so, but it is not uncommon for certain colleges to send their catalogues to students who are attending other colleges, and I even know of an instance where the "professor" of one college wrote a personal letter to the student of another college to secure his attendance. I know of another instance where the principal makes a practice of sending newspaper puffs of his institution to students and graduates and requests them to have them published in the papers. We are not allowed to place proprietary articles on the market, but the professors will give certificates and testimonials for proprietary and patented articles which are published in our professional journals. It is no wonder that veterinarians will occasionally go into business enterprises when they are set such examples by their tutors and preceptors.

It is easy to censure others for what we do not do, but before criticising others we should be careful that we are not guilty of acts of another kind just as liable to adverse criticism.

A college faculty without a respectable veterinary practice, and without means of imparting practical knowledge to its students, necessarily turns out graduates who are not competent to begin practice.

There are many graduates who utterly fail in practice simply because they have no practical knowledge to start with. There are many whose gross ignorance and glaring mistakes cause such an amount of opprobrium that it is difficult for competent men to overcome the effect of it. We are all liable to mistakes, I am sorry to say, but mistakes are made by many for which there can be but one excuse, and that is the inadequacy of the manner or method of the institutions that turn them out. Examples of this class of men are numerous, but I will give only a few instances: A graduate of a three-terms school endeavored for more than an hour to throw a colt to perform the ordinary operation of castration, and then allowed a non-graduate to castrate the colt for him. This graduate was examining for a live-stock insurance company, and in the course of his examination met with three or four horses with cataracts, when he communicated to me that he had discovered a new contagious disease of horses' eyes. He was treating a horse for what he diagnosed as "sweeney," and had the shoulder raw from repeated blisters, when another veterinarian was called in and pointed out a large ringbone as the cause of the lameness. Another graduate treated a valuable St. Bernard bitch for a year for what he diagnosed as "the womb out;" he gave the owner medicine which, he said, "would shrivel up the womb so that it would go back itself." The owner finally became discouraged and went to another veterinarian, who readily diagnosed the trouble as eversion of the vagina and removed the protrusion with a knife, and the bitch is now with pups. This same graduate was called to see a mare with relaxation of the sphincter ani; when she trotted the ingress and egress of air was accompanied by a noise. In explaining to the graduate the owner mentioned this noise as the annoying symptom. The graduate pulled off his coat, rolled up his sleeves, inserted his hand into the vagina, and, after more or less manœuvres, he informed the owner that the "mare's womb was down and a little twisted." He told the owner to inject "a teaspoonful of carbolic acid in a quart of water, and she would soon be all right." It was in vain the owner told him that she made the noise with the "upper hole."

Most of you are personally acquainted with the veterinary editor of an agricultural journal who, in an article intended to instruct farmers how to remove the placenta from a mare, got his anatomical knowledge ridiculously mixed and told them to unbutton the placenta from the cotyledons in the same manner they unbuttoned their coats. This same graduate recently reported a horse as having glanders, caused the horse to be locked up and the owner put to much trouble, simply because the horse had an offensive discharge from the nostril; another veterinarian was called in who demonstrated by trephining that the trouble was caused by a diseased tooth, with collection of pus in the sinus, and, as a consequence, the horse instead of being destroyed is now doing his daily work.

Such instances as the foregoing are not uncommon, and I do not mention them in ridicule, but in order to illustrate the point that such mistakes are due to a want of practical knowledge, and to emphasize the fact that if those graduates had received proper demonstrations at their schools such mistakes should not have occurred.

Of more importance than an increase in the number of terms is an increase in the amount of practical work at our schools. I am of opinion that it should be compulsory for students to serve, at least, as much time under qualified practitioners (in fact, an apprenticeship) as they are required to serve at college, and at their final examination they should be compelled to demonstrate in practical work that they are competent to practice.

All colleges should enforce a rigid examination to ascertain if an intending student has sufficient intelligence and sufficient education to comprehend lectures and instructions. It is not a rare thing to meet graduates whose attempts to write prescriptions are as disgusting as their ill-timed boasting of their diplomas and their science.

Another class of men we occasionally meet are those who have no idea of animal nature; they cannot even approach a horse without fear, and we see them stand at a safe distance from the animal when they should be at his side. They are too timid to pick up a horse's foot, and yet I have seen such men attempt to instruct a blacksmith how to shoe a horse.

Next to the inadequacy of colleges and lack of practical instruction is the lack of the adaptability of the individual. A veterinary graduate may know Latin, German, and French, the literature of the world may be open to him, he may be expert with the microscope and may be able to talk learnedly of pathology, histology, and bacteriology, he may be a first-rate office or library companion and a real clever fellow, but if he has no knack in handling stock and is afraid to go near a horse, the symptoms of an ordinary colic will temporarily scare out of his head all the scientific and linguistical lore he has accumulated. Such men made a mistake when they entered our profession, and their failure to succeed in practice cannot be charged to colleges.

In order to improve the standard of graduates we must have better equipped colleges, better qualified teachers, and I may truthfully say better qualified students, then we may expect better qualified graduates as a natural result. But an increase in the number of terms will not alone secure this result; an apprenticeship system will, however, greatly tend to it, as by this means the student will be enabled to learn the nature of the knowledge he has to acquire, as well as his fitness, ability, and his probabilities of success.

I hope our Association will not be too hasty in taking action on the question of three terms. For the present let us at least be conservative and just. I think you will find that the number of incompetent men graduated from three-terms schools are in proportion to those graduated from two-terms schools, of course taking into consideration the proportionate number each school graduates. The majority of the members of our Association were graduated from two-terms schools, and the eligible men in this State are so few that it will be much better for us to endeavor to create a standard among ourselves than to draw the line on the number of terms a man may have attended college. Our ranks are not overcrowded in this State, and let us hope the new-comers will be eligible to membership in this Association whether from two-terms or from three-terms schools.

To some of you it is known that there is a desire to start a veterinary college at Richmond, and you also know that I have been asked to head the effort, but I have never hesitated to discourage the idea on the ground that the proper material is lacking to make what I consider a veterinary institution should be.

In order to keep abreast with advanced ideas it is necessary for us to read our professional journals, and I believe we are all more or less liberal subscribers to those publications, and in this regard I desire to call your attention to the *Virginia Medical Monthly*, a journal worthy of our support. I will add that its editor requests for publication all papers that are of interest to the veterinary and medical professions combined. I will also say that if sufficient interest is taken in the matter by veterinarians the editor will include a veterinary department in his journal.

Membership in our Association is open to members of the medical profession, and I am glad to say that influential men of that profession are joining with us. Disease is disease, whether in man or beast, and the commingling of the representatives of the two professions at our meetings can only result in mutual benefit.

As the Committee on Legislation will make a report at this meeting, the subject will be generally discussed. For one, I am not in favor of being hasty in having laws passed. We should go slow, and whatever we ask for let it be reasonable, and let it be a foundation on which to build in the future. I think you will find that the majority favors asking for a law that will simply protect and define our titles.

I am unalterably opposed to any measure that will make legal practitioners of non-graduates with the right to assume our titles, no matter how long they have been in practice. While it is true there are some non-graduates who are worthy men and honestly do the best they can, there are also in this State hundreds of frauds and imposters, both white and black, who cannot even read or write, many of whom have been practising ten or twenty years, or longer, who would become recognized practitioners under any law that made it possible for a man to become a registered veterinarian simply because he could prove that he had been dosing horses for a specified number of years. The legal recognition of such men would be preposterous ; it would carry us back too many years. It is all well enough for the advocates of such measures to assert that it is the only means of getting rid of such men eventually, but you must bear in mind that we would not see the last of them in our days.

It would be much better in every way to have no law at all; much better to allow them to continue to practice as they are doing, without license and without standing or recognition. Simply have a law to prevent them from using our titles, and we will get rid of them sooner than by legalizing them in the manner done by some other States.

At present we cannot expect a law to confine the practice to legitimate veterinarians. Graduates in this State are too few to ask for such a measure with any chance of success. There are in Virginia one hundred counties, and according to our information only about ten counties have graduates. It would be waste of time to either formulate a bill or ask for one which will prevent the people of the other ninety counties from employing what they consider their best help when their animals are ill.

Personally, I am of the opinion that the only way to regulate the practice is by a State Board of Examiners, similar to those which regulate the practice of the medical, dental, and pharmaceutical professions in this State. It should be compulsory for every man to pass a satisfactory examination before the board before he could begin practice, whether a graduate or non-graduate. This, I think, is the best way to assure competent men, but the time has not yet arrived for us to ask for such a law. We must wait till we are stronger in numbers and influence and until the public generally becomes better acquainted with our profession.

From the report of the chairman of the Committee on Statistics you will learn the extent of the infectious and epizoötic diseases in the sections of the State where our members reside. This report alone is sufficient to convince anyone of the necessity of a State veterinarian. It also plainly indicates the necessity of better legislation on the contagious diseases of animals.

I have conversed with a number of persons since Dr. Niles's bill was submitted to me, and it is the general opinion that this State should have such a set of laws, but that it will be useless to ask for any legislation that will require an appropriation of money at the next session, on account of the condition of the treasury. However, I hope that the work on this bill will be continued and that it will be perfected in every possible way, so that when the occasion is ripe it will be in form for presentation, but I consider it useless to make the effort at the coming session.

It is probable that the next session will pass the bill to reorganize the State Board of Health, and if it does the prospects are good for at least one of the members being a legitimate veterinarian.

I think it advisable that we should at this meeting devise means of issuing in book or pamphlet form the proceedings of the Association. If there is not sufficient money in the treasury to cover the expense an extra assessment will not embarrass us. The records and papers even up to date are valuable and should be in a printed form for ready reference as well as for preservation. The Board of Censors, or sub-committee from it, should be empowered to attend to this subject and select such matter as in their judgment should be embodied in the publication. I sincerely hope that favorable action will be taken on this suggestion and that we will have our transactions in book form some time during this year.

The past year has been a fruitful one in regard to adding to the list of valuable veterinary literature. Translations and original works of merit have been placed within our reach which are of incalculable aid to the hard-working and earnest practitioner. Our thanks are especially due to Dr. W. L. Zuill for his translation of Freidberger and Fröhner's unequalled work on the *Pathology and Therapeutics of Domestic Animals*. Without any disparagement to time-honored authors like Williams, Fleming, and others, who will always be gratefully remembered, I do not think it extravagant to say that this translation of Freidberger and Fröhner's work gives to the English-reading veterinarian an opportunity to acquire a scientific and rational knowledge of his profession such as has never before been possible. It is really a wholesome revelation to the average modern veterinary college professor, and it is to be hoped that he will be benefited by it to the extent of at least absorbing some of its exact scientific teachings, so that he will be better fitted to impart instruction to his students.

Before concluding I wish to say that this address is meant for the welfare of our profession in this State, and that none of my remarks were prompted by malice, and I believe that many of the views I have expressed will be indorsed by the majority of the hard-working and earnest practitioners who are at present making the standard of the veterinarian on this continent.

In any community the profession is what we ourselves make it, and it is within our power to make much or little of it. The graduate should always bear in mind that he is the legitimate holder of the ancient and honorable title of veterinarian, once held and well used by no less a genius than Virgil himself. We must not consider ourselves the successors of the farriers; they were a class that held sway as a result, I may say, of vicious ignorance, and in their palmy days they did not even know enough to resurrect our title.

To those who aspire multiple titles or degrees, and to those who have the strange desire to affix half or more than half of the letters of the alphabet to their names, I simply recommend the study of the history of our profession as well as its principles and practice, with the hope that they will honor the title of veterinarian. The non-graduate is no discredit to the graduate, but the man with a diploma, if he is incompetent or does not consider our profession an honorable one, is a source of more harm to the profession than all other causes combined.

In conclusion, gentlemen, I thank you again for making me the first President of the Virginia State Veterinary Medical Association. I hope all who succed me in the office will appreciate the honor as I have done.

